# Phase II study of short-time oxaliplatin, capecitabine and epirubicin (EXE) as first-line therapy in patients with non-resectable gastric cancer

**DOI:** 10.1038/sj.bjc.6604569

**Published:** 2008-08-26

**Authors:** K R Schønnemann, H A Jensen, M Yilmaz, B V Jensen, O Larsen, P Pfeiffer

**Affiliations:** 1 Department of Oncology, Odense University Hospital, Odense C 5000, Denmark; 2 Department of Oncology, Herlev Hospital, Copenhagen, Denmark; 3 Department of Oncology, Ålborg Hospital, Copenhagen, Denmark

**Keywords:** advanced gastric cancer, oxaliplatin, capecitabine, epirubicin, EXE

## Abstract

Epirubicin, cisplatin and continuous infusion of 5-FU is a widely used palliative regimen in patients with gastric cancer. If cisplatin is substituted by oxaliplatin and 5-FU by capecitabine this regimen can be administered in the outpatient setting. Dose-limiting toxicity of oxaliplatin is peripheral sensory neuropathy and it is recommended to give oxaliplatin as a 120 min infusion. However, in patients with colorectal cancer a 30 min infusion of oxaliplatin can safely be administered without increasing neurotoxicity, standard infusion time is 30 min at our departments. In our phase I study the recommended doses of EXE was established (Dupont *et al*, 2006). Patients with non-resectable gastric adenocarcinoma were eligible. Patients received EXE (epirubicin 50 mg m^−2^ day 1; capecitabine 1000 mg m^−2^ day^−1^ continuously and oxaliplatin 130 mg m^−2^ day 1) as outpatient therapy every third week for a maximum of 8 cycles. From June 2004 to September 2005, we enroled 54 patients. Median age was 60 years (31–74 years) Median number of courses was 6 (1–8). Response rate was 45%. Median PFS was 6.8 (5.2–7.9) months and median survival was 10.1 (7.9–11.1) months. Most important grade 3 toxicities were as follows: nausea, vomiting, and diarrhoea (6%). Neurotoxicity grade 2 was seen in 36.5%. We therefore conclude, that EXE every third week is a convenient regimen that easily can be administrated in the outpatient setting but the regimen needs further evaluation in a phase III study.

Gastric cancer (GC) remains a significant global health problem and is one of the most frequent causes of cancer-related deaths. Although the incidence has been declining in Europe during the last century it is still the fifth most common cancer and an estimated 171 000 new cases of GC will be diagnosed each year (Boyle and Ferlay, 2005). The prognosis is very poor, with a 5-year overall survival of approximately 10% in Denmark (Coleman *et al*, 2003). The only potentially curative treatment for GC is surgery, but unfortunately most cases are diagnosed at an advanced stage because of very few organ-specific symptoms and therefore only about 20% of the patients are suitable for curative surgery (Janunger *et al*, 2001).

Therefore the need for palliative treatment is huge for these patients. The efficiency of palliative chemotherapy is widely accepted. Data from randomised studies have shown that combination chemotherapy results in a significant survival advantage and improves quality of life in patients with advanced GC (Murad *et al*, 1993; Pyrhonen *et al*, 1995; Glimelius *et al*, 1997; Wagner *et al*, 2006).

In the past decade a number of effective chemotherapy regimens have been established worldwide. However, these studies have not resulted in a standard regimen for gastric cancer, but the backbone of these regimens is most often a combination of 5-fluorouracil (5-FU) and platinum. In Europe ECF (epirubicin 50 mg m^−2^ day 1, cisplatin 60 mg m^−2^ day 1 and continuous infusion (CVI) of 5-FU 200 mg m^−2^ daily) is widely used as it was shown that ECF improves response rate and prolongs survival when compared with FAMtx which was considered to be a standard therapy a decade ago (Webb *et al*, 1997; Waters *et al*, 1999).

We sought to find a regimen that was comparable with ECF in efficacy, easy to administer in an outpatient setting and with low toxicity.

Therapy with 5-FU requires a central venous catheter which is associated with risk of venous thromboses and infection (Findlay *et al*, 1994; Evans *et al*, 1996). Capecitabine is an oral fluoropyrimidine, which is absorbed from the gastrointestinal tract as an intact molecule and afterwards converted into 5-FU in a cascade of three enzymatic steps (Miwa *et al*, 1998). Capecitabine is a well-established alternative which simplifies the administration of 5-FU and overcomes the problem with the central venous catheter.

Another cornerstone in ECF is cisplatin, which is highly emetic and in addition intravenous hydration is required to prevent renal toxicity.

Oxaliplatin is a third-generation platinum that easily can be administered in outpatients without hydration. When compared with cisplatin, oxaliplatin has a better safety profile with regard to renal toxicity and emesis. Recent studies have shown synergism between oxaliplatin and 5-FU and a combination of these two drugs has proven effective as first- or second-line treatment for advanced colorectal cancer (De Gramont *et al*, 2000; Raymond *et al*, 2001; Cassidy *et al*, 2004). The dose-limiting toxicity of oxaliplatin is peripheral sensory neuropathy, which is reversible but cumulative. Usually it is recommended to give oxaliplatin as a 120 min infusion to minimize peripheral neuropathy. However, we have recently in patients with colorectal cancer demonstrated that a 30 min infusion apparently does not result in more neurotoxicity (Pfeiffer *et al*, 2003, 2006).

We therefore used 30 min infusion of oxaliplatin in both our phase I and phase II trial (Dupont *et al*, 2006). The primary aims of our phase II study were to evaluate response rate and toxicity of EXE in patients with advanced gastric cancer. Secondary end points were progression-free survival and overall survival.

## Materials and methods

### Patient selection

Eligible patients were required to have histological verified non-resectable gastric or gastro-oesophageal junction adenocarcinoma and at least one measurable lesion according to RECIST criteria.

Eligibility criterias also included WHO performance status 0–2; age between 18 and 75 years; estimated life expectancy of at least 3 months; adequate hepatic function (serum bilirubin <1.5 × upper normal limit (UNL); transaminase <3 × UNL, however in cases of livermetastases, there was no upper limit for transaminases); adequate renal function (calculated creatinine clearance >30 ml min^−1^ by the Cockroft and Gault formula); adequate haematological function (neutrophil count >1.5 × 10^9^ l^−1^; platelets >100 × 10^9^ l^−1^ and no prior chemotherapy other than adjuvant chemotherapy, completed at least six months before inclusion.

Other inclusion criterias were ability to tolerate and comply with oral medication, no sign of peripheral neuropathy, no co-existent severe medical illness, no sign of brain metastases and no concomitant treatment with other anticancer therapy. Females were not included if they were pregnant or lactating.

The study was approved by the local ethics committee and Danish health authority and all participants gave written informed consent before entering the study.

### Study design and treatment

Patients were planned to receive a combination of epirubicin 50 mg m^−2^ as a 20 min. i.v. infusion day 1, oxaliplatin 130 mg m^−2^ as a 30 min. i.v. infusion day 1 and capecitabine 500 mg m^−2^ × 2 daily continuously each 3 weeks (Dupont *et al*, 2006).

Therapy was repeated every 3 weeks up to a maximum of eight cycles of treatment unless stopped before because of disease progression, unacceptable toxicity or patient refusal.

Reduction of 25% in all drug doses was recommended in the event of occurrence of febrile neutropenia, grade 4 thrombocytopenia (platelet count <25 × 10^9^ l^−1^) or grade 4 neutropenia (absolute neutrophil count <0.5 × 10^9^ l^−1^), grade 3–4 mucositis, diarrhoea, or nausea/vomiting in spite of optimal antiemetic treatment. Toxicity was graded according to NCIC-CTC version 2.0. Peripheral sensitive neuropathy to oxaliplatin was graded according to the following oxaliplatin-specific scale. Grade 1, parestesias/hypoesthesias of short duration with complete recovery before the next cycle; grade 2, parestesias/hypoesthesias persisting between two cycles without functional impairment; and grade 3 permanent parestesias/hypoesthesias resulting in functional impairment (Caussanel *et al*, 1990). In case of grade 2 neuropathy oxaliplatin was reduced 25%.

### Patient evaluation

Baseline evaluation included physical examination, assessment of medical history, evaluation of performance status and blood counts. During treatment patients were evaluated before each cycle of therapy with the above parameters. All patients had an abdominal and thoracic CT-scan performed at baseline and every third cycle to assess tumour response. Tumour response was classified according to RECIST guidelines and confirmed as lasting longer than 4 weeks.

After the end of the therapy patients were followed every third month until progression or death.

### Statisistical methods

The primary end point of this phase II study was response rate to EXE and secondary end points were progression-free survival (PFS), and overall survival (OS). Progression-free survival was defined as the time from inclusion to progressive disease occurred (according to the RECIST criteria) or death of any cause. Overall survival was defined as the time from inclusion to death of any cause. Progression-free survival and OS were updated until 1 September 2007.

Non-parametric statistics was applied. All median values are followed by range in brackets. After cessation of treatment patients without documented progression were followed every 3 months with clinical and radiological evaluation. Progression-free survival and OS were generated according to the Kaplan–Meier method. Data were recorded and analysed in a Medlog® database. All analyses were done on an intention-to-treat population.

## Results

### Patient charasteristics

Between June 2004 and August 2005, 54 patients with gastric and gastro-oesophageal adenocarcinoma were treated at three Danish oncology centres. Baseline patient characteristics are shown in Table 1. The median age was 59 years (range, 31–74 years). Performance status was 0 in 25 patients, one in 26 patients and two in three patients. No patients had previously received adjuvant chemotherapy or radiotherapy. Forty-three patients had adenocarcinoma located in cardia and 11 patients had adenocarcinoma located in the corpus or antrum of the stomach. Nine patients (17%) had locally advanced disease and 45 patients (83%) had metastatic disease. Twenty-four patients had more than one organ involved (range, 2–4).

### Toxicity

Fifty-three patients were evaluable for toxicity as one died of gastrointestinal bleeding 5 days after the first cycle of chemotherapy. Worst toxicity for all patients and all cycles are listed in Table 2. Neutropenia was the principal haematologic toxicity, 13% of the patients experienced grade 2 neutropenia, 15% grade 3 and 2% grade 4 neutropenia, but no patient had febrile neutropenia. Infection grade 2–3 without neutropenia was seen in 12%. In general grade 4 toxicity was rare, one patient experienced grade 4 neutropenia and one had vomiting in grade 4.

Non-haematologic toxicities were primary grade 2 toxicity, diarrhoea (13%), nausea (32%), vomiting (22%), hand food syndrome (PPE) (10%) and peripheral neuropathy (36%). No patients had peripheral neuropathy or PPE grade 3 or 4, and only 6, 4, and 6% had diarrhoea, vomiting or nausea grade 3, respectively. Except for the patient who died of gastrointestinal bleeding there was no treatment-related death in this study.

### Efficacy

The median number of EXE was 6 (range, 1–8), 19 patients completed eight cycles of treatment. The reasons for discontinuation of EXE were progressive disease or deterioration of health (*n*=21), toxicity (*n*=7), patient refusal (*n*=5) and other (*n*=2). One patient had complete response and 23 obtained partial response, giving an overall response rate of 45% in the ITT population.

**Figure 1:**
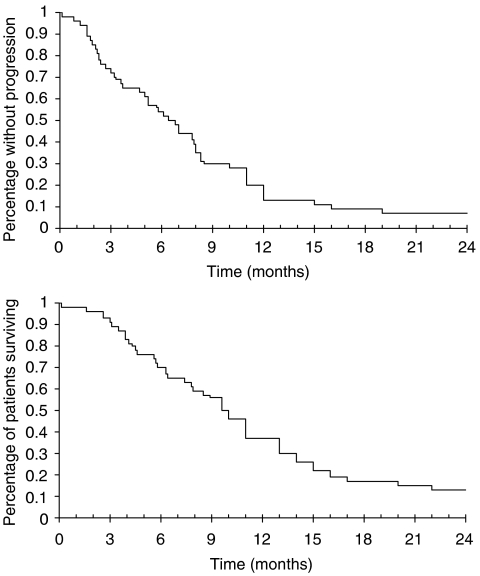
Time-to-progression and overall survival. Kaplan–Meier curves of time-to-progression (median 6.8 months; 95% CI 5.2–7.9 months) and overall survival (median 10.1 months; 95% CI 7.9–11.1 months).

Progression-free survival was 6.8 months (range, 5.2–7.9) and median survival was 10.1 months (range, 7.9–11.1) (Figure 1). Five patients are still alive 24–37 months after inclusion, one without active disease and four patients who have either had surgery or second- and third-line chemotherapy after progression.

## Discussion

Combination chemotherapy prolongs survival and improves quality of life in patients with advanced GC but still they have a poor prognosis and no standard regimen has yet been established.

In many areas of Europe, ECF has been regarded as a reference regimen in patients with advanced gastric or gastrooesophageal cancer as randomized studies have confirmed a high response rate around 45% and long overall survival around 9–10 months (Webb *et al*, 1997; Ross *et al*, 2002).

The introduction of the new anticancer drugs oxaliplatin and capecitabine made it possible to overcome problems with hydration and central venous catheter. A large randomized phase lll study, REAL-2, used ECF as control and substituted cisplatin with oxaliplatin and CVI 5-FU with capecitabine in a 2 × 2 factorial design. An impressive 1002 patients were enroled in REAL-2. Response rates were highest in the EOX group (47.9%) but did not differ significantly between the four groups. There was a nonsignificant difference in PFS among the groups, but there was a trend towards prolonged OS in patients receiving oxaliplatin and capecitabine EOX (Cunningham *et al*, 2008). In another phase III study, 220 patients were randomised to receive 24-h infusion 5-FU, leukovorin and cisplatin or 24-h infusion 5-FU, leukovorin and oxaliplatin (FLO). The authors also observed a trend towards a longer TTP in the FLO arm (median 5, 7 *vs* 3.8 months) (Al-Batran *et al*, 2006). Recently, a phase II study with 36 patients also evaluated a therapy with epirubicin, oxaliplatin and 5-FU. The response rate was 46%, PFS was 8.2 months and OS 12.2 months (Neri *et al*, 2007) Toxicity profile was very similar to the data in this study.

In recent years, ongoing or completed studies are evaluating new drugs or combinations of drugs in locally advanced or metastatic gastric cancer. Docetaxel, one of the ‘new’ drugs, was evaluated as first-line therapy in a phase III study (V325) with 457 patients receiving docetaxal, cisplatin and 5-FU (DCF) or cisplatin and 5-FU (CF). TTP (5.6 *vs* 3.7 months) and OS (9.2 *vs* 8.6 months) were significantly longer for patients receiving DCF (Van Custem *et al*, 2006). Docetaxel has also been used in combination with capecitabine, irinotecan and epirubicin in different phase II studies. An ongoing phase I and II study in five Danish oncology centres evaluates a combination of docetaxel, epirubicin and capecitabine. The primary end point for phase I is dose-limited toxicity. Primary end point for phase II study is response rate and secondary end points are PFS and OS.

The aims of the present phase II study, was to confirm the efficacy, convenience and tolerability of short-time EXE. We found a promising response rate of 46%, PFS of 6.8 months and OS of 10.1 months and all these efficacy data are comparable to the data observed in similar studies.

Short-time EXE is very well tolerated. Only seven patients (13%) stopped chemotherapy because of toxicity. Worst haematologic toxicity was neutropenia (grade 3–4 toxicity in 17 patients) but no patient had febrile neutropenia. Non-haematologic toxicity was primarily diarrhoea, nausea and vomiting but only two patients experienced nausea/vomiting grade 3–4. Despite the fact that oxaliplatin was infused in 30 min no patient developed peripheral neuropathy grade 3 and no patient experienced laryngopharyngeal dysaesthesia.

We therefore conclude, that a combination of epirubicin, capecitabine and oxaliplatin every third week is a convenient regimen that easily can be administrated in the outpatient setting but the regimen needs further evaluation in a phase III study.

## Figures and Tables

**Table 1:** Patient characteristics at baseline

**Characteristic**	***N***
*Age*	
Median	59
Range	31–74
	
*WHO performance status*	
0	25
1	26
2	3
	
*Location*	
Cardia	43
Corpus	11
	
*Prior adjuvant chemotherapy*	0
	
*No. of organs involved*	
1	30
2	17
>3	7
	
*Stage*	
Locally advanced disease	9
Metastatic disease	45
	
*Status of primary tumour*	
R0 resection	9
R1 resection	2
R2 resection	4
No surgery	39
	
*Increased alkaline phosphatase* (>300 U l^−1^)	4
	
*Increased ALAT* (0 U l^−1^)	8

**Table 2:** Worst toxicity after median 6 cycles of EXE

	**Grade 2 *n* (%)**	**Grade 3 *n* (%)**	**Grade 4/5 *n* (%)**
*Haematologic toxicity*			
Neutropenia	7 (13)	8 (15)	1 (2)
Thrombocytopenia	1 (2)	1 (2)	0
Infection (no neutropenia)	3 (6)	3 (6)	0
Febrile neutropenia	0	0	0
			
*Nonhaematologic toxicity*			
Diarrhoea	7 (13)	3 (6)	0
PPE	5 (10)	0	0
Nausea	17 (32)	3 (6)	0
Vomiting	12 (22)	2 (4)	1 (2)
Neuropathy	19 (36)	0	0
Bleeding			1 (2)
